# Chlorophyll a Fluorescence Transient and 2-Dimensional Electrophoresis Analyses Reveal Response Characteristics of Photosynthesis to Heat Stress in *Malus*. ‘Prairifire’

**DOI:** 10.3390/plants9081040

**Published:** 2020-08-15

**Authors:** Tao Wang, Siqian Luo, Yingli Ma, Lingyu Li, Yinfeng Xie, Wangxiang Zhang

**Affiliations:** 1 Co-Innovation Center for Sustainable Forestry in Southern China, College of Biology and the Environment, Nanjing Forestry University, Nanjing 210037, China; johnwt@cnbg.net (T.W.); lq8429996@gmail.com (S.L.); yli_ma@sina.com (Y.M.); li13505153082@sina.com (L.L.); wangxiangzh2002@sina.com.cn (W.Z.); 2 Institute of Botany, Jiangsu Province and Chinese Academy of Sciences (Nanjing Botanical Garden Mem. Sun Yat-Sen), Nanjing 210014, China

**Keywords:** heat stress, *Malus*. ‘Prairifire’, photosynthetic characteristics, chlorophyll a fluorescence, 2-dimensional electrophoresis

## Abstract

Flowering crabapples are a series of precious ornamental woody plants. However, their growth and development are inhibited in the subtropical regions due to the weak photosynthesis under high-temperature environment in the summer. Chlorophyll a fluorescence transient and 2-dimensional electrophoresis (2-DE) analyses were conducted to investigate the response characteristics of photosynthesis under simulated 38 °C heat stress in leaves of *Malus*. ‘Prairifire’, a spring-red leaf cultivar of flowering crabapple with strong thermal adaptability. In the present study, the net photosynthetic rate (Pn) was significantly decreased during the heat shock process, which showed a similar trend to the stomatal conductance (Gs), indicating a sensitive stomatal behavior to heat stress. Moreover, an efficient reaction center in photosystem II (PSII), and a functionally intact oxygen-evolving complex (OEC) conferred strong photosynthetic adaptability under heat stress. The higher level of transketolase (TK) under 48-h heat shock treatment was considered a protective mechanism of photosynthetic apparatus. However, heat stress inhibited the functions of light harvesting complex II (LHCII), electron transport in PSII, and the levels of key enzymes in the Calvin cycle, which were considered as the reasons causing an increase in the proportion of non-stomatal restrictions.

## 1. Introduction

Heat stress is one of the main environmental factors affecting plant growth and reproduction [1]. With the intensification of greenhouse gas emissions, extremely high temperatures occur frequently with longer duration, which is becoming a serious challenge for the entire agroforestry system worldwide [2].

As the basis of yield and quality in plants, photosynthesis is regarded as one of the most sensitive processes in response to heat stress [3] and has been reported to be the key to reveal the thermal adaptability of plants [4,5]. The impact of heat stress on photosynthesis can be mainly summarized as follows: in the early stage, heat stress causes the partial closure of stomata, which directly affects the net photosynthetic rate (Pn). Moreover, the changes in CO_2_ concentration caused by the decrease of the stomatal conductance (Gs) further limit the photosynthetic function [6]. Under strong heat stress, the membrane structure of the thylakoid would be damaged, resulting in a series of changes and even damages caused to structures and functions in photosystem II (PSII) [5], which is considered to be the most thermo-sensitive component of the photosynthetic apparatus [7].

Recently, in vivo chlorophyll a fluorescence rise (OJIP) kinetics has been applied extensively as a rapid and non-invasive tool for elucidating the activity of PSII in higher plants [3,8]. A quantitative analysis of the OJIP curves, called the JIP test, is used to reveal the environmental effect on the structure, conformation, and function of the photosynthetic organisms [8,9]. A large number of studies have proved the extreme sensitivity of the OJIP transient to heat stress, such as the L step (reflecting the energetic connectivity of the PSII units), the K step (relating to the inactivation of the oxygen-evolving complex (OEC)), and the J step, combining the changes of the IP phase (reflecting the status of Q_A_^−^ and electron transport from Q_A_ to Q_B_) [8,10]. Moreover, the photosynthetic activity in a PSII reaction center complex could be reflected by the index PI_ABS_, which is taken into consideration for three main functional steps, including light energy absorption, trapping of excitation energy, and conversion of the excitation energy to electron transport [3]. Therefore, research on the photosynthetic capacity of PSII based on OJIP curves has become an irreplaceable approach in heat-tolerance breeding in plants.

Moreover, proteomic approaches have provided important information for understanding the complex molecular mechanisms of plants in response to heat stress. Global protein expression profiles can be analyzed and compared using a two-dimensional gel-based protein separation method coupled with protein identification by mass spectrometry (MS). Among them, the expression and degradation of chloroplast proteins are regarded as necessary processes for normal growth and development in plants, as well as a common response mechanism to environmental changes. For example, the expressions of OEC and oxygen-evolving enhancer protein (OEE) can promote the absorption of light energy [11]. Magnesium chelatase plays an important role in maintaining the chlorophyll content and maximally absorbing light energy during chlorophyll synthesis [12]. Ferredoxin nicotinamide adenine dinucleotide phosphate (NADP) reductase can catalyze the synthesis of NADPH and thus plays an extremely important role in photosynthetic electron transport [13]. Moreover, a variety of enzymes involved in carbon assimilation, such as ribulose-1,5-bisphosphate carboxylase/oxygenase (rubisco), rubisco activase (RCA), phosphoribulose kinase, and transketolase (TK), have different abundances under heat stress, revealing the regulation of photosynthesis by chloroplast proteins [14,15].

Flowering crabapples (*Malus* spp.), members of Rosaceae, are a group of small landscape trees or shrubs with charming flowers, colorful fruits, and many tree shapes [16]. They originated in the temperate regions of northern China, and hundreds of varieties have been developed over the course of their more than 200-year history of cultivation, which are famous worldwide for their unique ornamental and economical values [17,18]. However, their cultivation in southern China, particularly within subtropical regions, is greatly limited because of the high-temperature environment in summer, which severely inhibits normal growth and development, especially photosynthesis [19]. *Malus*. ‘Prairifire’ is a spring-red leaf cultivar with excellent ornamental traits and strong thermal adaptability [20] and has passed the examination and approval of improved varieties of forest trees in Jiangsu Province (Su S-ETS-MP-007-2017). Therefore, *M*. ‘Prairifire’ is expected to be an ideal source for investigating the thermal adaption mechanism of crabapples. In the present study, chlorophyll a fluorescence combined with 2-dimensional electrophoresis (2-DE) analysis was used to study the response characteristics of photosynthesis to heat stress in leaves of *M*. ‘Prairifire’. The results of this study will provide a basis for the mechanism of heat resistance and genetic breeding of flowering crabapples. In addition, these results will provide scientific reference for the selection of subtropical landscape plants.

## 2. Materials and Methods

### 2.1. Plant Materials and Growth Conditions

Samples of one-year-old *M*. ‘Prairifire’ seedlings were selected from the arboretum of the national repository of *Malus* spp. germplasm (Yangzhou City, Jiangsu Province, China) in November 2015 and were transferred separately into individual plastic pots (23 cm tall × 30 cm diameter; one plant per pot), filled with prepared soil. The soil used in this experiment was yellow-brown soil collected from the arboretum and was homogenized, air-dried, and sieved through a 4.0-mm sieve. The soil was neutral (pH = 6.68), mixed with suitable organic fertilizer, containing 6.79 g organic matter, 1.86 g nitrogen, 127 mg available phosphorus, 295 mg available potassium per kg soil, and less than 1 mg mercury per kg soil. A total of 40 sample pots were placed in the arboretum for culturing, and the fully expanded, healthy leaves from the third branch (from the top) of the seedlings were marked for future experiments.

### 2.2. Treatments

In May 2016, uniform and healthy potted samples were transferred to an artificial climate chest, with the light intensity set as 600 μmol·m^−2^·s^−1^, under a light/dark cycle of 12/12 h, and 70–75% relative humidity at a temperature of 26 ± 1 °C for controlled growth (CG). 500 mL water was added to per pot every 2 days until the end of all the measurements. In June 2016, potted samples grown in the artificial climate chest were randomly arranged for artificial heat shock treatment. The heat shock temperature was set as 38 °C during 6:00 am–6:00 pm, which referred to a local average temperature under extreme high temperature conditions; while that was 26 °C during 6:00 pm–6:00 am. The other settings were the same as the CG conditions. The photosynthetic gas exchange parameters and chlorophyll a fluorescence transient were measured under the CG conditions and heat shock treatments for 1 day, 2 days, 4 days, and 6 days. The time of per measurement was under 9:00 am–11:00 am. Moreover, fresh leaves under CG conditions and those treated with 38 °C heat shock for 48 h were collected, quickly placed in liquid nitrogen for freezing, and then saved under −80 °C conditions for 2-DE analysis.

### 2.3. Measurements of Photosynthetic Gas Exchange Parameters

The photosynthetic gas exchange parameters of leaves of *M*. ‘Prairifire’ were measured using an LI-6400 portable photosynthesis system (Li-Cor, USA), with a 6 cm^2^ leaf chamber. The setting value of photosynthetically active radiation (PAR) was 600 μmol m^−2^ s^−1^ with a red–blue LED light source (6400-02B). The gas flow rate was 500 μmol s^−1^. The air temperature and relative humidity were consistent with the settings in the artificial climate chest. The measured parameters included the Pn, Gs, intercellular CO_2_ concentration (Ci), and transpiration rate (Tr). Six marked leaves were measured, and the data were recorded when the rate of CO_2_ uptake was stable. Finally, the average was calculated as the final result for each measurement time.2.4. Measurements of the chlorophyll a fluorescence transient

The chlorophyll a fluorescence transient for the leaves of *M*. ‘Prairifire’ was measured under CG conditions and on days 1, 2, 4, and 6 after the heat shock treatment. The leaves were dark-adapted for 30 min before the measurement; the OJIP curve was then determined using PEA-Senior (Hansatech, UK) and was analyzed using the JIP test, according to the methods of Strasser et al. [8]. Details of the introduced parameters are listed below: φP_O_ (*F_V_/F_M_*), maximum quantum yield of primary PSII photochemistry; Vj, the variable fluorescence at 2 ms; Sm, the normalized area (assumed to be proportional to the number of reduction and oxidation of one Q_A_^−^ molecule during the fast OJIP transient and therefore related to the number of electron carriers per electron transport chain); N, the times Q_A_ was reduced to Q_A_^−^ in the time span from t_0_ to t_Fmax_; ABS/RC, average absorbed photon flux per PSII reaction center; DI_O_/RC, the specific energy fluxes per reaction center for dissipation; TR_O_/RC, the specific energy fluxes per reaction center for trapping; ET_O_/RC, the specific energy fluxes per reaction center for electron transport; φE_O_, the probability that an absorbed photon will move an electron into the electron transport chain; PI_ABS_, the performance index of PSII; Ψ_O_, efficiency with which a trapped exciton can move an electron into the electron transport chain; and RC/CS_O_, the number of active PSII reaction centers per excited cross section.

### 2.4. Extraction and Quantification of Proteins in Leaves of M. ‘Prairifire’

1 g leaf samples were collected and ground in liquid nitrogen. The powder was transferred to a 50-mL centrifuge tube and dissolved by pre-cooled 10% TCA-acetone solution (containing 0.1% DTT and 1 mM PMSF) at −20 °C overnight. The supernatant was discarded by centrifugation at 15,000× *g* at 4 °C for 20 min, and a pre-cooled acetone solution (containing 0.1% DTT and 1 mM PMSF) was added to the precipitate at −20 °C for 2 h. Then, the supernatant was discarded by centrifugation at 15,000× *g* at 4 °C for 20 min, and the precipitate was placed in a freeze vacuum dryer for 30 min. The dried protein powder was stored in a −80 °C freezer for later use.

Protein powder (100 μg) was redissolved in 800 μL pyrolysis buffer (7 M urea, 2 M thiourea, 4% CHAPS, 65 mM DTT, 0.5% Bio-Rad Ampholyte, 1 mM PMSF). Then, the supernatant was retained by centrifugation at 15,000× *g* at 4 °C for 10 min. The quantification of proteins was performed using a Bradford Method Protein Concentration Assay Kit, according to the manufacturer’s instructions.

### 2.5. 2-DE Analysis

The sample buffer that was loaded with 100 µg protein and mixed with 400 μl pyrolysis buffer was applied to IPG adhesive strips (24 cm; pH 4–7; non-linear) for isoelectric focus (IEF). Mineral oil was used to prevent exposure to the air. The IEF procedure was set to 50 V for 14 h, 100 V for 1 h, 500 V for 1 h, 1000 V for 1 h, and 8000 V for 4 h, and the total voltage time product was 12000 V/h. After the end of IEF, the IPG strip was preserved in a 10-mL SDS equilibrium solution 1 (containing 6 M urea, 2% SDS, 0.375 M Tris-HCl, 20% glycerin, 1% DTT) and then subjected to a shaker balance for 15 min. Subsequently, the strip was cleaned, SDS equilibrium solution 2 was added (additional 2.5% IAA to equilibrium solution 1 without 1% DTT), followed by the use of the shaker balance for 15 min. After equilibrium was achieved, the IPG strip was placed above the 12% uniform polyacrylamide gel, and 10-µL protein marker was added to the SDS-polyacrylamide gel. The second dimension was performed until the bromophenol blue reached the bottom edge of the gel. The temperature of the cold cycle was set to 16 °C. After protein fixation in 40% methanol and 5% phosphoric acid for 1 h, the gels were stained with Coomassie brilliant blue G-250 for 20 h. The gels were then washed by distilled water, scanned in the Ettan DIGE Imager (GE Healthcare, Buckinghamshire, UK), and converted to electronic files, which were then analyzed using PDQuest software (Bio-Rad, Hercules, CA, USA).

### 2.6. Protein Identification and Database Search

Significant differentially expressed protein (DEP) was identified with a fold change ≥ 2 and a *p*-value ≤ 0.05. The DEP spots observed by 2-DE analysis were cut from the gel and washed by distilled water. Then, these DEP spots were destained by 100 mmol/L NH_4_HCO_3_ and were washed by 50% acetonitrile for 5 min. Next, the obtained DEP spots were added to 100% acetonitrile for 5 min, then digested by 5 µL 50 mM NH_4_HCO_3_ containing 10 ng Trypsin at 37 °C for 16 h. Subsequently, 20 µL NH_4_HCO_3_ was used to cover the tube. Then, 5% TFA was added for 10 min to stop the reaction. The dissolved samples mixed with saturated matrix HCCA at 1:1 were loaded onto the target instrument, dried, and subjected to MALDI-TOF-TOF-MS detection. The obtained mass fingerprint data were searched in the NCBInr database, and the search engine was Mascot.

### 2.7. Protein Annotation and Interaction

The identified protein ID was converted to UniProt ID. The identified proteins were then mapped to the Gene Ontology (GO) database by protein UniProt ID. Unannotated proteins were annotated by using Inter ProScan software through the protein sequence alignment method. Then, all annotated proteins were classified into three categories: biological process, cellular component, and molecular function.

The pathway of the identified protein was then annotated by the Kyoto Encyclopedia of Genes and Genomes (KEGG) database. The KEGG online service tool KAAS was used to annotate the protein’s KEGG description. Then, all annotated proteins were mapped to the KEGG pathway using the KEGG online service tool KEGG mapper. Moreover, wolfpsort, an updated version of PSORT/PSORT II, was used to predict the subcellular localization of all identified proteins.

All identified proteins were searched against the STRING database version 10.0 for protein-protein interactions (PPI). The interaction confidence was determined by a metric “confidence score” defined by STRING. The interaction network from STRING was visualized in Cytoscape.

### 2.8. Statistical Analysis

Statistical analysis was conducted using SPSS software version 19.0 and Microsoft Excel 2010. Multiple comparison analyses were performed using one-way analysis of variance (ANOVA) with Duncan’s test (*p* < 0.05). Microsoft Excel 2010 and PhotoShop CS6 were used to draw the plots.

## 3. Results

### 3.1. The Response of Photosynthetic Gas Exchange Parameters of M. ‘Prairifire’ Leaves Exposed to High Temperature Stress

**Figure 1:**
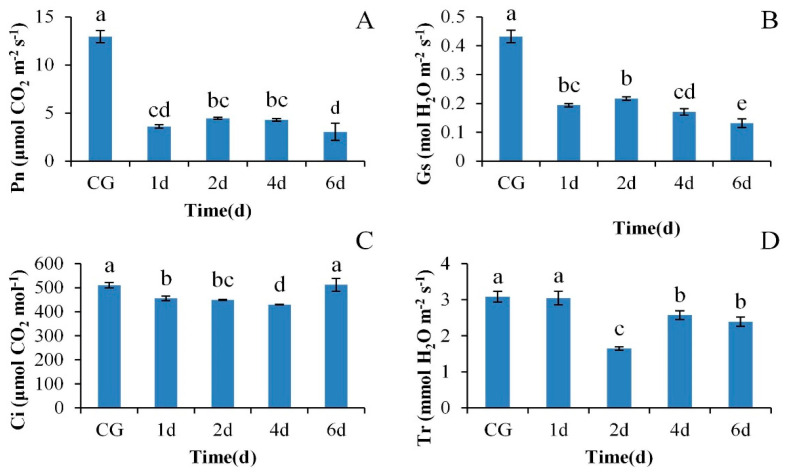
The changes of gas exchange parameters in Pn (**A**), Gs (**B**), Ci (**C**), Tr (**D**) in *M.* ‘Prairifire’ leaves exposed to 38 °C heat stress. Values are means ± SE (*n* = 6). Different lowercase letters indicate a significant difference at the 0.05 level between different treatments. CG: Plants were treated with CG conditions; 1d: Plants were treated with 38 °C heat shock for 1 day; 2d: Plants were treated with 38 °C heat shock for 2 days; 4d: Plants were treated with 38 °C heat shock for 4 days; 6d: Plants were treated with 38 °C heat shock for 6 days.

As Figure 1A shows, the value of Pn under CG treatment was 12.95 mol CO_2_·m^−2^·s^−1^, while 38 °C heat shock for 1 day caused Pn to drop by 72.14% (*p* < 0.05). Then, the Pn showed a trend of increasing first and then decreasing from day 2 to day 6 under heat shock treatment, and the Pn value on day 6 was significantly lower than that on day 4 (*p* < 0.05). The variation of Gs was consistent with that of Pn (Figure 1B). The lowest value of Gs was present on day 6 under heat shock, which was significantly different from that of Gs at other times (*p* < 0.05). The values of Ci showed a trend of decreasing first and then increasing (Figure 1C), and the lowest value of Ci was present on day 4 under heat shock. It was noteworthy that the Ci value on day 6 increased by 19.33% compared with that on day 4 (*p* < 0.05), showing an opposite trend to that of Pn and Gs. A significant decrease of 46.52% in Tr occurred on day 2 under heat shock treatment compared with that on day 1 (*p* < 0.05). Then, the Tr increased significantly (*p* < 0.05) and finally reached 2.39 mmol m^−2^·s^−1^ on day 6 of the heat shock treatment (Figure 1D).

### 3.2. The Response of OJIP Curves of M. ‘Prairifire’ to Heat Stress

**Figure 2:**
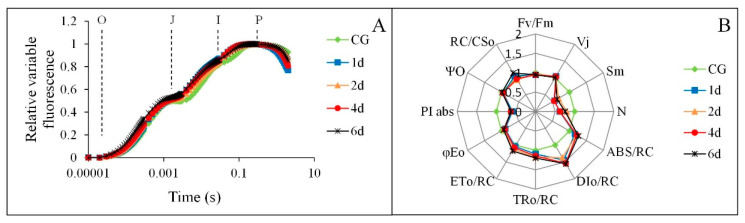
Effects of 38 °C heat stress on chlorophyll a fluorescence transient (**A**) and chlorophyll a fluorescence parameters (**B**) in *M.* ‘Prairifire’ leaves. Data are each the mean of 6 independent measurements. CG: Plants were treated with CG conditions; 1d: Plants were treated with 38 °C heat shock for 1 day; 2d: Plants were treated with 38 °C heat shock for 2 days; 4d: Plants were treated with 38 °C heat shock for 4 days; 6d: Plants were treated with 38 °C heat shock for 6 days.

As Figure 2A shows, the fluorescence rise kinetics of control plant samples exhibited a typical O-J-I-P shape. The fluorescence rise kinetics in leaves exposed to heat stress for 1–6 days still kept a whole O-J-I-P polyphasic transient curve. The heat shock did not cause significant changes in J, and P step, nor did it show significant K step, indicating that the function of OEC, reduction of Q_A_^−^ and fluorescence yield were not significantly affected. However, the increase of JI and IP phase may increase the burden of electron transport chain. Moreover, a large amount of information about the donor side, the acceptor side, and the reaction center of PS II was also provided by the JIP test (Figure 2B). Among these variables, Fv/Fm showed a slight decrease under 38 °C heat shock (*p* > 0.05), while PI_ABS_ was significantly reduced under the same condition (*p* < 0.05). Sm and N showed a fluctuating trend with a large variation range, which was significantly different from CG (*p* < 0.05). φEo showed similar variation to Sm and N, but only on day 1 of the heat shock was there a significant difference (*p* < 0.05). Higher values of TR_O_/RC and ET_O_/RC were present on day 2 and 6, respectively, under heat shock, which were significantly different from CG (*p* < 0.05). The variation of DI_O_/RC was basically consistent with TR_O_/RC and ET_O_/RC, and the values of DI_O_/RC during the heat shock process were significantly higher than those of CG (*p* < 0.05). Moreover, Vj increased slightly at first and then decreased gradually, which was opposite to Ψ_O_. RC/CS_O_ increased slightly on day 1 of heat shock, decreased significantly on day 2 (*p* < 0.05), and then gradually increased (*p* < 0.05). ABS/RC showed a rising trend under the heat shock process, and the values of ABS/RC during the heat shock process were significantly higher than those of CG (*p* < 0.05).

### 3.3. Identification of DEPs in Leaves of M. ‘Prairifire’ between CG and Shock Treatment for 48 h

**Figure 3:**
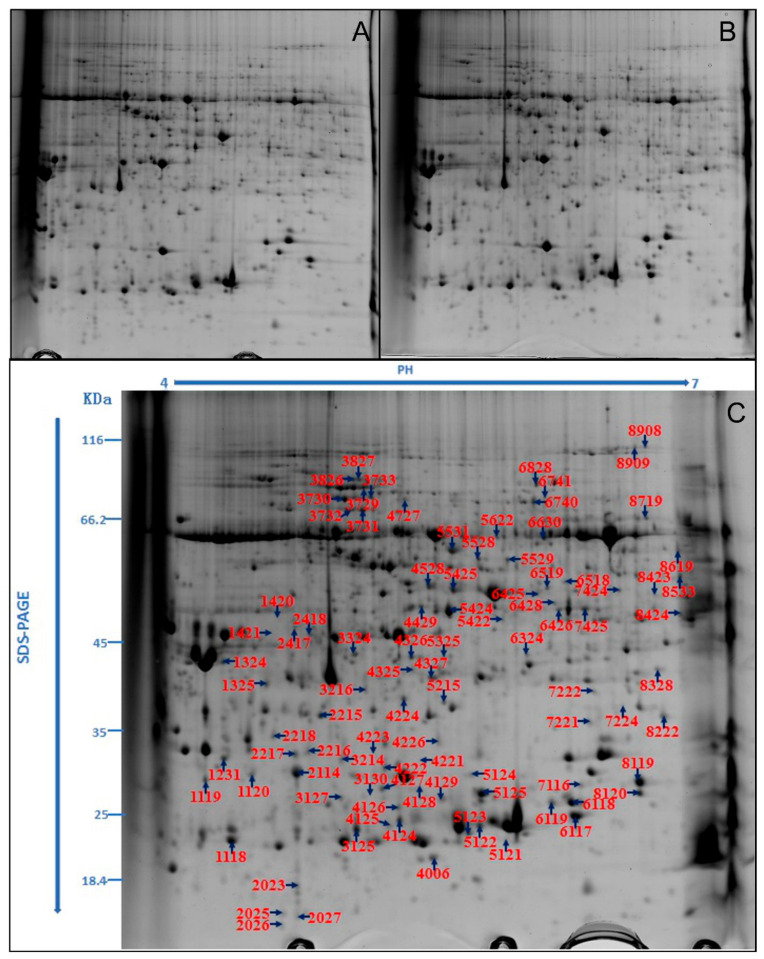
Representative 2-DE profiles of total proteins extracted from *M.* ‘Prairifire’ leaves treated with CG (**A**), and 38 °C heat shock for 48 h (**B**). (**C**) Representative 2-DE profiles of differential expressed proteins extracted from *M.* ‘Prairifire’ leaves between CG conditions and 38 °C heat shock for 48 h.

The representative maps of 2-DE in leaves of *M*. ‘Prairifire’ under CG and 48-h heat shock treatments are shown in Figs. 3A and 3B, respectively. The protein spots that showed large repetitive changes through automatic detection, matching, and manual editing were considered to be DEPs. A total of 38 reliable DEP spots were detected, including 19 that were down-regulated and 19 that were up-regulated (Figure 3C). All the DEP spots were extracted and identified by mass spectrometry. The obtained data were blasted with the sequence of known proteins in the NCBInr database, and 36 protein spots were identified successfully by excluding the same proteins. Therefore, compared with CG, 19 DEGs were down-regulated and 17 DEGs were up-regulated in leaves of *M*. ‘Prairifire’ under heat shock treatment for 48 h (Table S1).

**Figure 4:**
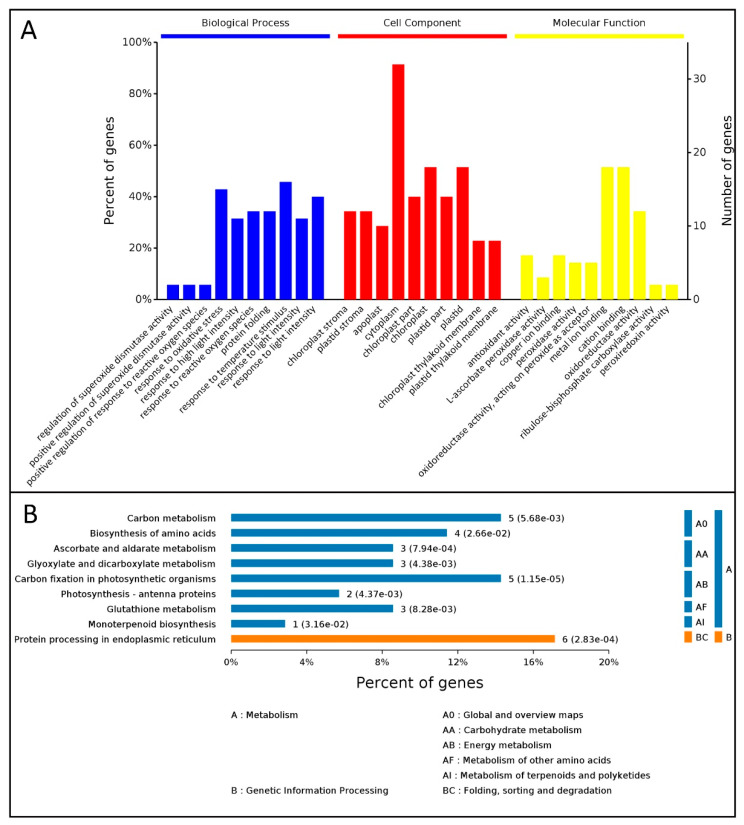
The function classification of GO (**A**) and KEGG pathway annotation (**B**) in *M*. ‘Prairifire’ leaves after 38 °C heat shock for 48 h.

The GO terms representing all the DEPs in leaves of *M*. ‘Prairifire’ under heat shock treatment for 48 h were classified into three categories: cellular component, molecular function, and biological process (Figure 4A). In the biological process, classification, oxidative stress response, and temperature stimulus response accounted for a large proportion. The cytoplasm, chloroplasts, and plastids were the most important components in the classification of cell components. In the molecular functional classification, metal ion binding and cationic binding were the main functions.

Moreover, 36 DEPs were classified into various KEGG pathways, of which nine metabolic pathways were significantly enriched (*p* < 0.05) (Figure 4B). In these metabolic pathways, more proteins were enriched in the endoplasmic reticulum, followed by carbon metabolism and carbon fixation in photosynthetic organics.

### 3.4. PPI Network for the DEPs in Leaves of M. ‘Prairifire’ between CG and Heat Shock Treatment for 48 h

**Figure 5:**
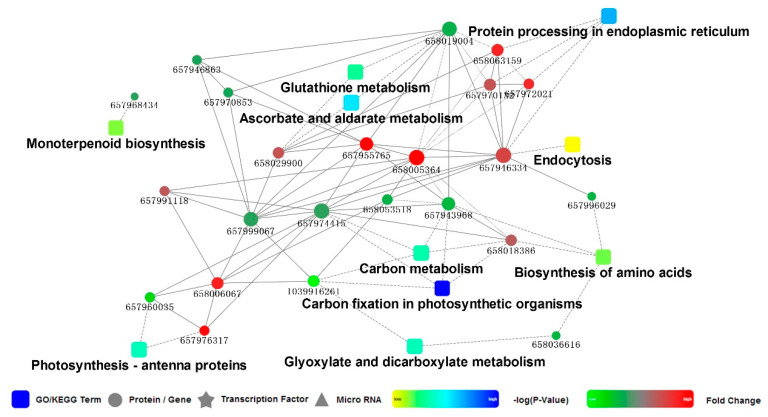
PPI speculation in *M.* ‘Prairifire’ leaves after 38 °C heat shock for 48 h.

The PPI network for the DEPs in leaves of *M*. ‘Prairifire’ between CG and heat shock treatment for 48 h was mapped to understand the dynamic changes in metabolic pathways and to generate hypotheses about the relationship between DEPs (Figure 5). In the present study, the enriched pathways of the PPI network were mainly involved in the endoplasmic reticulum, carbon fixation in the photosynthetic apparatus, and photosynthetic antenna proteins. The predicted interactions of proteins mainly included heat shock proteins, oxidoreductase, and chloroplast-related proteins. Interestingly, there was a highly reliable protein interaction between 20 kDa chloroplastic chaperonin proteins (CPN20) and several chloroplast proteins, such as TK, ATP-dependent zinc metalloproteinase FTSH2 (FTSH2), and glyceraldehyde-3-phosphate dehydrogenase A (GADPH), and between CPN20 and several oxidoreductases as well as heat shock proteins (HSPs), including HSP17.6 and HSP18.1 (Table S2).

### 3.5. Differential Expression of Photosynthesis-Related Proteins in Leaves of M. ‘Prairifire’ between CG and Heat Shock Treatment for 48 h

In the present study, a total of 14 photosynthesis-related proteins with different expressions under heat stress were obtained (Table 1). Most of these proteins were predicted to be located in the chloroplast. Among these proteins, the PS I chlorophyll a/b binding protein 3-1 (LHCA3), TK, OEE1, two CPN20 proteins, and a chloroplastic small HSP (HSP21) were up-regulated. Among these, LHCA3 and a CPN20 protein were more highly expressed under heat stress, i.e., 8.559 and 11.158 times higher, respectively, than in CG. The remaining proteins, including rubisco, ribulose diphosphate carboxylase long chain (rbcL), chlorophyll a-b binding protein of LHC II type 1 (LHCB1), 2-cysteine peroxidase BAS1 (2-Cys), FTSH2, GADPH, and thioredoxin H (Trx-H), were down-regulated. The differential expressions of the proteins mentioned above were involved in the light and dark reactions in the photosynthetic process, which indicated a unique regulation in response to heat stress.

## 4. Discussion

Photosynthesis, one of the main means for plants to obtain energy, is very sensitive to heat stress [21]. Our results showed that heat stress induced a significant decrease in Pn in the leaves of *M.* ‘Prairifire’, and with the extension of heat shock duration, Pn was further inhibited. According to the variation of gas exchange parameters in the present study and the judgment basis proposed by Farquhar and Sharkey [22], stomatal limitation was considered to be an important factor that resulted in the decrease of Pn under heat stress, which indicated a sensitive stomatal behavior in response to heat stress. Combined with the changes of Tr during the heat shock process, the partial closure of stomata may provide a protective strategy for maintaining water homeostasis in leaves of *M*. ‘Prairifire’ in response to heat stress, that is, maintaining a strict transpiration rate to ensure nearly constant leaf water potential and relative water content [23]. However, after long-term exposure (6 days) to heat shock, the opposite trend of Ci and Pn or Gs implied an increased proportion of nonstomatal restriction, which indicated that the mesophyll cell activity of *M*. ‘Prairifire’ decreased gradually. To reveal the underlying mechanisms of photosynthesis in *M*. ‘Prairifire’ responses to heat stress, chlorophyll a fluorescence transient combined with 2-DE analysis were performed, and the findings are discussed below.

### 4.1. Electron Transport Chain and Related Proteins Involved in the Light Reaction

LHCs are major constituents of the antenna systems in higher plant photosystems. The peripheral antennas of PSII are composed of major trimeric and minor monomeric LHCII proteins [24], while four LHCA subunits are tightly bound to the PSI core complex, forming its outer antenna moiety called LHCI. In the present study, the expression of LHCB1 was downregulated in *M*. ‘Prairifire’ under heat stress, which showed an opposite trend to that of LHCA3, indicating that LHCII in leaves of *M*. ‘Prairifire’ was more susceptible than that of LHCI to heat stress. LHCII proteins are switched from efficient light-harvesting state to photoprotective state in response to the changes of external environmental conditions. The reversible state transitions could balance the distribution of excitation energy between PSII and PSI, and are required for the functionality of PSI. Therefore, the thermal sensitivity of LHCII in the leaves of *M*. ‘Prairifire’ may be a protective mechanism to prevent damage to PSI, which is arguably harder to recover from heat stress injury. Moreover, a significant decrease in Sm and N and a gradual decrease in φEo in leaves of *M*. ‘Prairifire’ under heat stress were measured by the JIP test, suggesting that the function of the acceptor side in PSII was inhibited under heat stress, which could be attributed to the loss of the PQ pool, and the restraint of electron transport with respect to Q_A_, the primary quinone electron acceptor of PSII [8,25]. Therefore, the blocking of electron transport of PSII may become one of the main nonstomatal reasons for the decrease of photosynthetic ability in *M*. ‘Prairifire’ under heat stress.

However, from the JIP test, the performance of K steps under heat stress showed rare changes compared with that of CG, indicating functionally intact OEC in leaves of *M*. ‘Prairifire’ under heat stress. OEE is a key structural component of many different types of OECs and functions to stabilize the manganese cluster and to modulate the Ca^2+^ and Cl^−^ requirements for oxygen evolution [26]. Therefore, the upregulation of OEE1 in this study under heat stress could explain the activity of OEC under heat stress. Moreover, the level of Vj and Ψ_O_ showed little changes under heat treatment compared with the CG, indicating that the opening ratio of the active reaction center was less affected by the heat stress. With the extension of the heat shock process, ABS/RC, RC/CS_O_, and TR_O_/RC increased gradually, further indicating that heat stress increased the efficiency of energy utilization and consumption in the active reaction center [27,28]. These combined results showed an efficient reaction center in leaves of *M*. ‘Prairifire’ in response to heat stress. The improvement of heat dissipation and the efficiency of the active reaction center could explain the adaptive mechanism of the PSII reaction center under heat stress, combined with the changes of DI_O_/RC and ET_O_/RC [29]. Interestingly, the significant variations of PI_ABS_ between CG and heat shock treatments were more sensitive than those of Fv/Fm, indicating that PI_ABS_ could be selected as a key indicator reflecting the changes of the photosynthetic apparatus in *M*. ‘Prairifire’ under heat stress.

### 4.2. Proteins Involved in the Dark Reaction and Regulatory Mechanisms

Rubisco is a key functional protein involved in the dark reaction process of plants and can act as an oxygenase involved in catalyzing the first step of the plant photorespiration pathway. Moreover, it is also a carboxylase that mediates CO_2_ assimilation [30]. In the present study, the levels of rubisco and rbcL under heat shock treatment were down-regulated, indicating that carbon assimilation in the leaves of *M*. ‘Prairifire’ was significantly inhibited due to heat stress. Moreover, the levels of GADPH and phosphotriose isomerase were down-regulated after 48 h of heat shock, further indicating that the Calvin cycle of *M*. ‘Prairifire’ leaves was inhibited, which may be the main reason contributing to the increased proportion of non-stomatal limitations under heat stress. Previous studies have reported oxidative damage caused to lipids, proteins, and even DNA by heat stress [31]. In the present study, the down-regulation of 2-cys and Trx-H in *M*. ‘Prairifire’ implied a risk of gradual accumulation of reactive oxygen species (ROS) in chloroplasts [31,32,33]. The down-regulation of FTSH2 further indicated a decrease of the ability to degrade photooxidatively damaged products of the D1 protein [34]. Therefore, one of the possible reasons for the down-regulation of photosynthetic regulatory protein levels under heat stress was attributed to the accumulation of ROS leading to oxidative stress.

However, TK was detected to be highly expressed after heat shock treatment, indicating that the regeneration ability of ribulose 1, 5 diphosphate were enhanced [35]. Besides of the regulation of carbon fixation in higher plants, TK is also reported to be involved in the response of higher plants to abiotic stress, thus enhancing their stress resistance [36,37,38]. Weber reported that increased TK activity could alter photosynthate allocation in favor of sucrose biosynthesis, and regulate the flux into phenylpropanoid metabolism [39], which conferred added heat resistance to photosynthetic apparatus. Therefore, the thermal stability of the photosynthetic metabolism in leaves of *M*. ‘Prairifire’ was probably due to the high level of TK, which is considered as a regulation of photosynthetic adaptability. Interestingly, according to the PPI results from STRING, CPN20 was predicted to have a highly reliable interactions with a series of photosynthesis-related proteins, such as TK and HSP21. Studies have proved the functions of chloroplastic HSPs that improve the thermotolerance of plants [40]. As a unique co-chaperonin in higher plant chloroplasts, CPN20 has also been reported to be involved in the folding of specific client proteins by forming chaperonin systems [41]. Therefore, the high level of photosynthetic proteins such as TK in the leaves of *M*. ‘Prairifire’ under heat stress may be results of interacting with CPN20. The expression and interaction verification of related proteins is expected to be the key to further reveal the heat resistance of *M*. ‘Prairifire’.

## 5. Conclusions

The assessment of the results allows us to conclude that heat stress significantly inhibited Pn in leaves of *M*. ‘Prairifire’ through both stomatal and non-stomatal limitations. According to the changes of gas exchange parameters, a sensitive stomatal behavior in *M*. ‘Prairifire’ may be a protective strategy for maintaining water homeostasis under heat stress. Chlorophyll a fluorescence combined with 2-DE analysis was conducted to illustrate the response mechanism of photosynthetic apparatus to heat stress. The increased proportion of non-stomatal factors under heat stress could be derived from the sensitivity of LHCII to heat stress, the inhibition of electron transport in PSII, and the down-regulated expression of key enzymes in the Calvin cycle. It was noteworthy that, the photosynthetic apparatus of *M*. ‘Prairifire’ demonstrated a functionally intact OEC, which could be explained by higher levels of OEE1, and an efficient reaction center in PSII under heat stress. These combined results could responsible for strong photosynthetic adaptability in *M*. ‘Prairifire’ leaves. Moreover, the high level of TK under heat stress was regarded as a regulatory mechanism to provide some protection to the photosynthetic apparatus against heat stress. Interestingly, CPN20 was predicted from STRING to have a reliable interaction with TK, indicating a possible protective effect that improves the expression of photosynthetic proteins in chloroplasts under heat stress.

## Figures and Tables

**Table 1:** Differential expressed photosynthesis-related proteins in leaves of *M*. ‘Prairifire’ between CG and heat shock treatment for 48 h.

Number	Protein Name	gi Number	UniProt ID	Fold Change
1	PREDICTED: thioredoxin H-type	gi|657970853	P29448	0.445
2	PREDICTED: triosephosphate isomerase, cytosolic-like	gi|657943968	P48491	0.292
3	PREDICTED: 2-Cys peroxiredoxin BAS1, chloroplastic-like	gi|657999067	Q9C5R8	0.486
4	PREDICTED: ATP-dependent zinc metalloprotease FTSH 2, chloroplastic	gi|658053518	O80860	0.344
5	PREDICTED: chlorophyll a-b binding protein of LHCII type 1	gi|657960035	Q39142	0.143
6	PREDICTED: ribulose bisphosphate carboxylase large chain	gi|1039916261	O03042	0.059
7	ribulose-1,5-bisphosphate carboxylase/oxygenase large subunit, partial (plastid)	gi|817992125	O03042	0.243
8	PREDICTED: glyceraldehyde-3-phosphate dehydrogenase A, chloroplastic	gi|657974415	Q9LPW0	0.498
9	PREDICTED: photosystem I chlorophyll a/b-binding protein 3-1, chloroplastic-like	gi|657976317	Q9SY97	8.559
10	PREDICTED: small heat shock protein, chloroplastic	gi|657970132	P31170	2.356
11	PREDICTED: 20 kDa chaperonin, chloroplastic-like	gi|658005364	O65282	11.158
12	PREDICTED: 20 kDa chaperonin, chloroplastic-like	gi|657947332	O65282	2.415
13	PREDICTED: transketolase, chloroplastic, partial	gi|658018386	F4IW47	2
14	PREDICTED: oxygen-evolving enhancer protein 1, chloroplastic	gi|658006067	Q9S841	5.688

## Supplementary Materials

The following are available online at https://www.mdpi.com/2223-7747/9/8/1040/s1, Table S1. The mass spectrometry identification of differential expressed proteins in leaves of *M*. ‘Prairifire’ between CK and heat shock treatment for 48 h; Table S2. PPI score.
