# Supplementary material for: Chlorophyll a Fluorescence Transient and 2-Dimensional Electrophoresis Analyses Reveal Response Characteristics of Photosynthesis to Heat Stress in Malus. ‘Prairifire’
Source: Plants (Basel). 2020 Aug 15;9(8):1040. doi: 10.3390/plants9081040 (PMC7464964; doi:10.3390/plants9081040)
Supplement: Supplementary file 1 [file plants-09-01040-s001.zip › supplementary files/List of supplementary files.docx]

**List of supplementary files**

Tab. S1 The mass spectrometry identification of differential expressed proteins in leaves of *M.* ‘Prairifire’ between CK and heat shock treatment for 48 h.

Tab. S2 PPI score
