# Supplementary material for: Chlorophyll a Fluorescence Transient and 2-Dimensional Electrophoresis Analyses Reveal Response Characteristics of Photosynthesis to Heat Stress in Malus. ‘Prairifire’
Source: Plants (Basel). 2020 Aug 15;9(8):1040. doi: 10.3390/plants9081040 (PMC7464964; doi:10.3390/plants9081040)
Supplement: Supplementary file 1 [file plants-09-01040-s001.zip › supplementary files/Tab.S1.docx]

Tab S1 The mass spectrometry identification of differential expressed proteins in leaves of *M*. ‘Prairifire’ between CK and heat shock treatment for 48 h

| protein number | MS number | gi number | UniProt ID | fold change | protein name | score | nominal mass (Mr) | calculated pI value | peptides number | Sequence Coverage |
| --- | --- | --- | --- | --- | --- | --- | --- | --- | --- | --- |
| 1325 | H21 | gi\|657962836 | Q8VZJ2 | 0.309 | PREDICTED: glucan endo-1,3-beta-glucosidase-like | 240 | 37404 | 5.03 | 2 | 8% |
| 2023 | G09 | gi\|658049152 | Q93VR4 | 0.491 | PREDICTED: major allergen Pruav 1-like | 164 | 17557 | 5.37 | 3 | 28% |
| 2025 | J13 | gi\|657970853 | P29448 | 0.445 | PREDICTED: thioredoxin H-type | 209 | 14068 | 5.56 | 2 | 22% |
| 2114 | J15 | gi\|657943968 | P48491 | 0.292 | PREDICTED: triosephosphate isomerase, cytosolic-like | 283 | 27605 | 5.75 | 4 | 24% |
| 2218 | H12 | gi\|657999067 | Q9C5R8 | 0.486 | PREDICTED: 2-Cys peroxiredoxin BAS1, chloroplastic-like | 205 | 29521 | 7.75 | 3 | 13% |
| 3125 | H14 | gi\|145581388 | Q05431 | 0.343 | ascorbate peroxidase | 101 | 27712 | 5.53 | 2 | 9% |
| 3127 | G13 | gi\|658019004 | Q05431 | 0.362 | PREDICTED: L-ascorbate peroxidase, cytosolic-like | 181 | 27700 | 5.67 | 3 | 16% |
| 3324 | G15 | gi\|657968434 | Q9M2E2 | 0.484 | PREDICTED: (+)-neomenthol dehydrogenase | 185 | 32379 | 5.2 | 3 | 14% |
| 3733 | J06 | gi\|658053518 | O80860 | 0.344 | PREDICTED: ATP-dependent zinc metalloprotease FTSH 2, chloroplastic | 300 | 74158 | 5.74 | 2 | 6% |
| 4125 | J05 | gi\|657960035 | Q39142 | 0.143 | PREDICTED: chlorophyll a-b binding protein of LHCII type 1 | 232 | 28241 | 5.29 | 2 | 13% |
| 4127 | H06 | gi\|657946863 | Q9XEX2 | 0.457 | PREDICTED: peroxiredoxin-2B-like | 213 | 17550 | 5.23 | 3 | 18% |
| 4429 | I09 | gi\|657971219 | P52577 | 0.488 | PREDICTED: isoflavone reductase-like protein | 278 | 34050 | 5.72 | 4 | 14% |
| 5121 | I21 | gi\|1039916261 | O03042 | 0.059 | PREDICTED: ribulose bisphosphate carboxylase large chain | 241 | 29623 | 6.1 | 3 | 10% |
| 5425 | H11 | gi\|658036616 | Q56WN1 | 0.302 | PREDICTED: glutamine synthetase cytosolic isozyme | 202 | 39096 | 5.78 | 3 | 9% |
| 5528 | J04 | gi\|817992125 | O03042 | 0.243 | ribulose-1,5-bisphosphate carboxylase/oxygenase large subunit, partial (plastid) | 79 | 50013 | 6.46 | 2 | 3% |
| 5529 | I07 | gi\|657996029 | P23686 | 0.311 | PREDICTED: S-adenosylmethionine synthase 2-like | 170 | 43465 | 5.67 | 4 | 13% |
| 8120 | I17 | gi\|658045245 | Q4PSY4 | 0.496 | PREDICTED: uncharacterized protein LOC103422041 | 170 | 43465 | 5.67 | 4 | 13% |
| 8423 | I08 | gi\|657974415 | Q9LPW0 | 0.498 | PREDICTED: glyceraldehyde-3-phosphate dehydrogenase A, chloroplastic | 187 | 43250 | 8.1 | 3 | 10% |
| 8909 | G08 | gi\|6969976 | Q9LHA8 | 0.484 | high molecular weight heat shock protein | 299 | 71570 | 5.17 | 6 | 7% |
| 2217 | K01 | gi\|657976317 | Q9SY97 | 8.559 | PREDICTED: photosystem I chlorophyll a/b-binding protein 3-1, chloroplastic-like | 90 | 29925 | 7.85 | 1 | 3% |
| 3214 | J16 | gi\|657970132 | P31170 | 2.356 | PREDICTED: small heat shock protein, chloroplastic | 162 | 24664 | 7.88 | 3 | 13% |
| 3729 | G20 | gi\|657946334 | Q9LHA8 | 3.019 | PREDICTED: heat shock 70 kDa protein isoform X1 | 121 | 71796 | 5.22 | 3 | 5% |
| 4126 | H17 | gi\|657964030 | P29830 | 2.983 | PREDICTED: 17.1 kDa class II heat shock protein-like | 155 | 17484 | 5.56 | 2 | 12% |
| 4326 | H22 | gi\|658029900 | Q1PER6 | 2.341 | PREDICTED: L-ascorbate peroxidase 2, cytosolic | 101 | 27887 | 5.39 | 1 | 8% |
| 4528 | G05 | gi\|657971221 | Q9S7T8 | 2.183 | PREDICTED: serpin-ZX-like | 135 | 47749 | 6.63 | 2 | 7% |
| 5122 | G21 | gi\|657972021 | P29830 | 4.892 | PREDICTED: 17.9 kDa class II heat shock protein-like | 129 | 17434 | 5.94 | 2 | 25% |
| 5125 | H19 | gi\|658063159 | P19037 | 5.351 | PREDICTED: 18.1 kDa class I heat shock protein-like | 129 | 17897 | 5.58 | 3 | 19% |
| 5325 | I13 | gi\|657991118 | O80934 | 2.133 | PREDICTED: uncharacterized protein At5g02240-like | 172 | 35095 | 9.16 | 3 | 9% |
| 6117 | I14 | gi\|658005364 | O65282 | 11.158 | PREDICTED: 20 kDa chaperonin, chloroplastic-like | 498 | 26285 | 8.55 | 5 | 24% |
| 5215 | G11 | gi\|657947332 | O65282 | 2.415 | PREDICTED: 20 kDa chaperonin, chloroplastic-like | 275 | 26348 | 7.74 | 4 | 15% |
| 6118 | G07 | gi\|657955765 | O81235 | 10.241 | PREDICTED: superoxide dismutase [Mn], mitochondrial | 136 | 25855 | 7.92 | 3 | 14% |
| 6740 | G23 | gi\|658039957 | Q9ZVS4 | 2.572 | PREDICTED: basic 7S globulin-like | 392 | 47572 | 5.77 | 4 | 12% |
| 6828 | G12 | gi\|658018386 | F4IW47 | 2 | PREDICTED: transketolase, chloroplastic, partial | 94 | 72577 | 5.78 | 2 | 3% |
| 7116 | J23 | gi\|658003653 | Q9STS7 | 2.718 | PREDICTED: temperature-induced lipocalin-1-like | 151 | 21471 | 6.18 | 2 | 13% |
| 7224 | G24 | gi\|658006067 | Q9S841 | 5.688 | PREDICTED: oxygen-evolving enhancer protein 1, chloroplastic | 459 | 35263 | 5.76 | 5 | 16% |
| 7424 | J01 | gi\|384563685 | none | 2.815 | polyphenol oxidase V | 97 | 68009 | 5.67 | 2 | 3% |
